# Conoidin A, a Covalent Inhibitor of Peroxiredoxin 2, Reduces Growth of Glioblastoma Cells by Triggering ROS Production

**DOI:** 10.3390/cells12151934

**Published:** 2023-07-26

**Authors:** Monika Szeliga, Radosław Rola

**Affiliations:** 1 Department of Neurotoxicology, Mossakowski Medical Research Institute, Polish Academy of Sciences, 5 Pawińskiego Str., 02-106 Warsaw, Poland; 2 Department of Neurosurgery and Paediatric Neurosurgery, Medical University of Lublin, 8 Jaczewskiego Str., 20-090 Lublin, Poland; rola.radoslaw@gmail.com

**Keywords:** peroxiredoxin, glioblastoma, adenanthin, conoidin A, reactive oxygen species, autophagy

## Abstract

Compounds that cause oxidative stress have recently gained considerable interest as potential anticancer treatment modalities. Nevertheless, their efficiency may be diminished by the antioxidant systems often upregulated in cancer cells. Peroxiredoxins (PRDXs) are antioxidant enzymes that scavenge peroxides and contribute to redox homeostasis. They play a role in carcinogenesis and are upregulated in several cancer types. Here, we assessed the expression pattern of PRDX1 and PRDX2 in glioblastoma (GBM) and examined the efficacy of their inhibitors in GBM cell lines and patient-derived GBM cells. Both PRDX1 and PRDX2 were upregulated in GBM compared to non-tumor brain tissues and their considerable amounts were observed in GBM cells. Adenanthin, a compound inhibiting PRDX1 activity, slightly decreased GBM cell viability, while conoidin A (CONA), a covalent PRDX2 inhibitor, displayed high toxicity in GBM cells. CONA elevated the intracellular reactive oxygen species (ROS) level. Pre-treatment with an ROS scavenger protected cells from CONA-induced death, indicating that ROS accumulation plays a crucial role in this phenomenon. Menadione or celecoxib, both of which are ROS-inducing agents, potentiated the anticancer activity of CONA. Collectively, our results unveil PRDX1 and PRDX2 as potential targets for GBM therapy, and substantiate the further exploration of their inhibitors.

## 1. Introduction

Gliomas are a histologically and molecularly heterogeneous group of neoplasms, accounting for 80% of malignant primary brain tumors. Glioblastoma (GBM; WHO IV) is the most frequent adult primary brain tumor. Despite the progress made in our understanding of biology of glial tumors over the past decade, the clinical outcome of patients with GBM still remains poor: the median survival time is less than one year [1]. Diffuse brain infiltration, high rates of cell proliferation, heterogeneity, and the presence of a subpopulation of self-renewing GBM stem-like cells (GSCs) contribute to resistance to treatment [2].

Increasing evidence indicates that oxidative stress is linked to GBM tumorigenesis, although it is still unclear whether an imbalance in the redox state is a cause or an effect of this process [3]. GBM cells produce large amounts of reactive oxygen species (ROS) which affect the cell cycle and are involved in tumor progression and resistance to treatment. ROS-induced damage in DNA, RNA, proteins, and lipids leads to genomic instability, enhancing cancer development. Both radio- and chemotherapy cause an increase in intracellular ROS which trigger apoptosis. Several novel compounds that destroy cells through the formation of intracellular ROS have been recently examined as potential anti-glioma drugs. However, GBM cells adapt to elevated ROS levels by activating an extended antioxidant cellular response [3,4]. Similarly to the other types of neoplastic cells, GBM cells undergo the upregulation of antioxidant enzymes, such as manganese superoxide dismutase (MnSOD), thioredoxin (TRX), thioredoxin reductase (TrxR), catalase (CAT), and superoxide dismutase (SOD), which helps them to escape cell death under conditions of oxidative stress [5,6,7].

Peroxiredoxins (PRDXs) belong to a family of six thiol-dependent peroxidases. Their primary role is to reduce the peroxides in water, which protects the cell from peroxide toxicity. Besides this antioxidant function, PRDXs play signal regulatory roles in various signaling networks [8]. A growing body of evidence points to the importance of these enzymes in carcinogenesis. Depending on the tumor type and the stage of progression, PRDX1 can either inhibit or promote cancer growth [9,10,11,12]. The remaining five PRDX isoforms are considered to be tumor promoters [12,13,14,15,16,17]. These findings suggest that compounds targeting PRDXs may exert anticancer properties. Indeed, adenanthin (ADNT), which has been shown to inhibit the antioxidant activity of PRDX1 and, to a lesser extent, PRDX2 [18], displays cytotoxic effects in leukemic and hepatocellular carcinoma cells [18,19]. Conoidin A (CONA), a covalent PRDX2 inhibitor [20], reduces the viability of gastric cancer cells [21].

Data on the expression pattern of PRDX proteins in GBM are scarce and inconsistent. Immunohistochemical analysis of PRDX levels in a large cohort of astrocytic brain tumor tissues revealed that the majority of the astrocytic tumors were positive for PRDX1 and PRDX2, and their expression decreased significantly with an increasing malignancy grade [22]. However, according to Odreman et al., PRDX1 was upregulated in a few GBM cases, but barely detectable in healthy brain tissues [23]. The silencing of *PRDX1* increases the chemo- and radiosensitivity of glioma cells in vitro [24], while also prolonging survival in mouse glioma models [25]. Moreover, a decreasing *Prdx2* expression sensitizes rat glioma cells to oxidative stress [26].

In the current study, we set out to examine the levels of both PRDX1 and PRDX2 in GBM and non-tumor (NT) brain tissues. Subsequently, we evaluate the efficacy of ADNT and conoidin A (CONA) in GBM cell lines and patient-derived GBM cells. To the best of our knowledge, the activity of neither of these two compounds has been analyzed in GBM models so far.

## 2. Materials and Methods

### 2.1. Sample Collection

GBM tissues were collected from 41 patients who underwent surgical resection in the Department of Neurosurgery and Paediatric Neurosurgery, Medical University of Lublin, Poland. All patients gave their written informed consent for use of tissues in research. All procedures were in accordance with the Declaration of Helsinki and approved by the local Ethics Committee. All tissues were examined by pathologist according to the WHO criteria [27]. Twenty NT brain samples were obtained from The Netherlands Brain Bank (NBB), Netherlands Institute for Neuroscience, Amsterdam (open access: www.brainbank.nl). All materials were collected from donors, for or from whom a written informed consent for a brain autopsy and the use of the material and clinical information for research purposes were obtained by the NBB.

### 2.2. Cell Culture and Handling

In this study, three human GBM cell lines were used: T98G (ATCC, Manassas, VA, USA), U87MG (Sigma-Aldrich, St. Louis, MO, USA) and LN229 (kindly provided by Dr. Rafał Krętowski, Department of Pharmaceutical Biochemistry, Medical University of Białystok, Poland). T98G cells were grown in MEME (Sigma-Aldrich) containing 10% fetal bovine serum (FBS) (Gibco, Thermo Fisher Scientific, Grand Island, NY, USA) and non-essential amino acids (Gibco). U87MG cells were cultured in EMEM (ATCC) supplemented with 15% FBS. LN229 cells were maintained in DMEM (Gibco) containing glucose and 10% FBS.

To establish patient-derived cultures, tumor specimens were mechanically and enzymatically dissociated and filtered through 100 μm cell strainers. Cells were resuspended in DMEM/HAM’s F12 medium (Gibco) supplemented with either 10% FBS for adherent cultures, or B-27 (1:50 dilution), epidermal growth factor (20 ng/ mL), and basic fibroblast growth factor (10 ng/mL) for sphere cultures. All the above supplements were obtained from Gibco.

Normal human astrocytes (NHAs) (ScienCell Research Laboratories, Carlsbad, CA, USA) were cultured in the astrocyte medium as recommended by the manufacturer.

All media were supplemented with penicillin and streptomycin purchased from either Gibco or ScienCell Research Laboratories. The cells were kept at 37 °C in a humidified atmosphere of 5% CO_2_ and 95% air.

### 2.3. Chemicals

Conoidin A was purchased from Cayman Chemical (Ann Arbor, MI, USA). Adenanthin (ADNT) was obtained from ChemFaces (Wuhan, China). N-acetylcysteine (NAC), menadione (MEN) and celecoxib (CCX), and were obtained from Sigma-Aldrich. Dimethyl sulfoxide (DMSO) (Sigma-Aldrich) was used as vehicle and its final concentration did not exceed 0.2% *v*/*v*.

### 2.4. Gene Expression Analysis

RNA was isolated using TRI-Reagent (Sigma-Aldrich). The RNA concentration was determined by using the NanoDrop2000 spectrophotometer (NanoDrop Technologies Inc., Wilmington, DE, USA). A High-Capacity cDNA Reverse Transcription Kit (Applied Biosystems, Thermo Fisher Scientific, Waltham, MA, USA) was used to synthesize cDNA. The quantitative real-time PCR reactions were carried out using the TaqMan Universal PCR Master Mix (Applied Biosystems, Thermo Fisher Scientific) and the primers obtained either from Applied Biosystems (PRDX1, cat. No.: Hs00602020_mH; PRDX2, cat. No.: Hs03044902_g1) or Blirt (Gdansk, PL) (*ACTB* coding for β-actin, cat. No.: HK-DD-hu). The expression of either *PRDX1* or *PRDX2* was normalized to the expression of *ACTB* and the ΔΔCT method [28] was used to calculate the relative expression.

### 2.5. Immunoblotting

Tissue or cell lysates were prepared in RIPA buffer (Sigma-Aldrich). A bicinchoninic acid (BCA) assay (Pierce, Rockford, IL, USA) was used to quantify total protein. Subsequently, proteins were separated by SDS-PAGE and transferred to PVDF membranes. After blocking with 5% non-fat dried milk for 1 h at RT, the membranes were incubated with primary antibodies overnight at 4 °C, which was followed by incubation with a secondary antibody conjugated with horseradish peroxidase (HRP) for 1 h at RT. The chemiluminescence signal of immunoreactive proteins was obtained using the SuperSignal West Pico Chemiluminescence Substrate (Pierce). Images were visualized and captured with the G-Box gel documentation system (Syngene, Frederick, MD, USA) or on X-ray film. Densitometric quantification of the bands was performed with ImageJ 1.53e software (NIH, Bethesda, MD, USA).

The following primary antibodies were purchased from ProteinTech (Chicago, IL, USA): anti-PRDX1 (#15816-1-AP), anti-PRDX2 (#10545-2-AP), anti-β-actin (#81115-1-RR), and anti-GAPDH (#HRP-60004). The antibody against LC3 (#L7643) and the secondary anti-rabbit antibody (#A0545) were obtained from Sigma-Aldrich.

### 2.6. MTT Cell Viability Assay

Cell viability was measured with a 3-(4,5-dimethylthiazol-2-yl)-2,5-diphenyl tetrazolium bromide (MTT) assay. The cells (4 × 10^4^ cells per well) were seeded in 24-well plates (#662160; Greiner Bio-One, Kremsmünster, Austria) and incubated overnight. Subsequently, they were exposed to increasing concentrations (0, 25, 50, and 100 μM) of ADNT, increasing concentrations (0, 1, 5, 10 and μM) of CONA, 5 μM of MEN, 25 μM of CCX, or their combinations for 72 h. To study the effect of ROS on the viability, 5 mM NAC was administered for 1 h before the addition of CONA. Next, an MTT (Sigma-Aldrich) solution was added (final concentration of 0.5 mg/mL). After 2 h of incubation formazan crystals were dissolved in DMSO, and absorbance was measured at 570 nm using an Elisa Bio-Rad Microplate Reader (Bio-Rad, Hercules, CA, USA). In a single experiment, each concentration of compounds or their combinations were analyzed in triplicates.

### 2.7. Colony Formation Assay

Two protocols were used to perform this assay. In the first protocol, cells were seeded in 6-well plates (#657160; Greiner Bio-One) in culture medium (2 × 10^3^ of T98G, U87MG, and LN229 cells per well or 3 × 10^3^ of LUB17 and LUB20 cells per well) and incubated for 24 h (37 °C, 5% CO_2_). The following day, cells were treated with increasing concentrations (0, 1, 5, and 10 μM) of CONA and allowed to grow for 72 h. After this time, the medium was changed, and cells were allowed to grow in drug-free medium for 10–14 days. In the second protocol, cells were first treated with increasing concentrations (0, 1, 5, and 10 μM) of CONA for 72 h, and then were washed with PBS, trypsinized, and counted. Subsequently, 2 × 10^3^ cells were seeded in 6-well plates in CONA-free medium and cultured for 10 days. Then, colonies were fixed with 4% formaldehyde for 10 min, stained with 0.5% crystal violet solution in 25% methanol for 15 min, rinsed with deionized water to remove any residual dye, and air-dried at RT. The grossly visible colonies were counted manually.

### 2.8. Formation of Neurospheres

LUB17N cells were seeded in 24-well plates (#662160; Greiner Bio-One) pre-treated with the anti-adherence rinsing solution (#07010; Stemcell Technologies, Vancouver, Canada). After 24 h, cells were treated with increasing concentrations (0, 1, 5, and 10 μM) of CONA, 5 μM of MEN, 25 μM of CCX, or their combination for 20 days. Next, spheres were observed using an Axiovert 40C inverted phase-contrast microscope (Zeiss, Oberkochen, Germany) and images were randomly taken from each condition. Number and size of neurospheres were analyzed using Image J software.

### 2.9. Measurement of Intracellular ROS

Intracellular ROS levels were measured using the fluorescent dye 2′,7′-dichlorodihydrofluorescein diacetate (H2DCFDA) (Sigma-Aldrich). The cells were seeded in 96-well plates (#3596; Corning, Glendale, CA, USA), cultured overnight, and treated with CONA for 24 h. After this time, the cells were loaded with 10 μM of H2DCFDA and incubated at 37 °C for 30 min. Next, the fluorescence intensity was measured at a 488 nm excitation wavelength and 525 nm emission wavelength using a microplate reader (Infinite M2000, Tecan, Männedorf, Switzerland).

### 2.10. Statistical Analysis

Data are expressed as mean ± SD from at least three independent experiments. The statistical analysis was performed using GraphPad Prism 7.0 (San Diego, CA, USA). The statistical significance was determined by either a Mann–Whitney test, Student’s *t* test (for comparison between two groups), or by one-way analysis of variance (ANOVA) (for comparisons between more than two groups).

## 3. Results

### 3.1. Both PRDX1 and PRDX2 Are Upregulated in GBM Tissues and Cells

**Figure 1:**
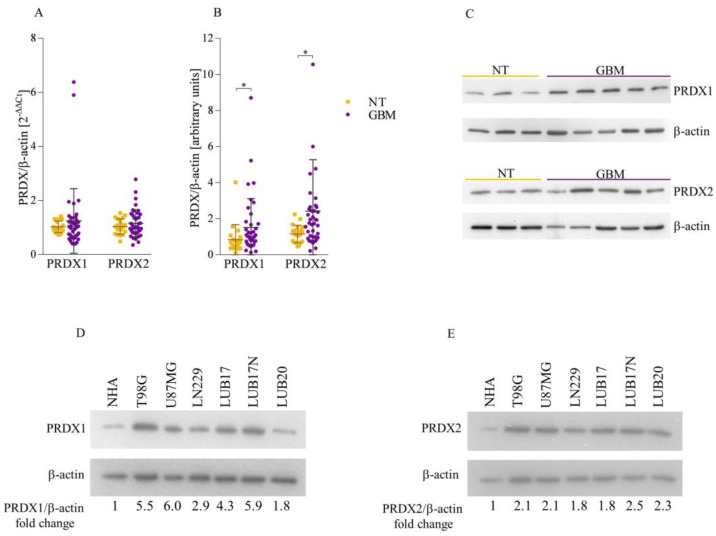
The level of both PRDX1 and PRDX2 is elevated in GBM tissues and cells. (**A**) The expression of *PRDX1* and *PRDX2* in NT (n = 20) and GBM (n = 41) tissues. (**B**) Densitometric analysis of PRDX1 and PRDX2 protein bands (relative to β-actin) in NT (n = 20) and GBM (n = 41) tissues. Statistical analysis was performed with Mann–Whitney U test. * *p* < 0.05. (**C**) Representative blots showing the level of PRDX1 (upper panel) and PRDX2 (lower panel) in NT and GBM tissues. β-actin was used as a loading control. (**D**,**E**) Representative Western blot results showing the level of PRDX1 (**D**) and PRDX2 (**E**) proteins in NHAs and T98G, U87MG, LN229, LUB17, LUB17N, and LUB20 cells. β-actin was used as a loading control. Bands were quantified by densitometry, determining the quotient of the densitometry signal for PRDX1 or PRDX2 band, and that for β-actin was calculated and then normalized to that of the NHAs. Average fold-change values from three independent protein isolations are shown.

To gain deeper insight into the expression pattern of *PRDX1* and *PRDX2* in brain tumors, we first examined the level of each of these isoforms in NT and GBM tissues. Both *PRDX1* and *PRDX2* expression was slightly elevated in GBM compared to NT specimens (Figure 1A). Furthermore, Western blot analysis revealed markedly higher levels of PRDX1 and PRDX2 proteins in GBM than in NT tissues (Figure 1B,C and Figure S1). In further studies, we used human GBM cell lines T98G, U87MG, and LN229, as well as two patient-derived cell cultures grown as adherent monolayers, hereinafter referred to as LUB17 and LUB20, respectively. Additionally, LUB17 cells were cultured as neurospheres and termed LUB17N. We observed the high upregulation of PRDX1 and PRDX2 proteins in GBM cells compared to NHAs (Figure 1D,E). Overall, the above results demonstrate considerable amounts of PRDX1 and PRDX2 in GBM tissues and cells.

### 3.2. Impact of ADNT and CONA on Viability of GBM Cells and NHAs

**Figure 2:**
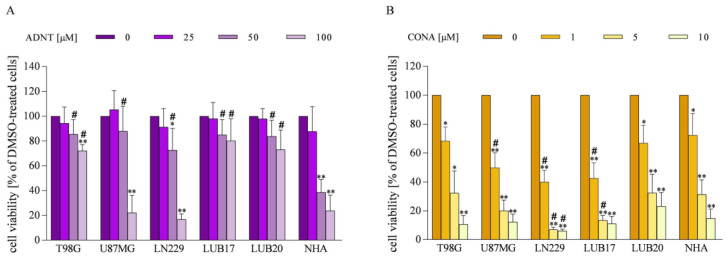
Cytotoxic effects of ADNT or CONA in GBM cells and NHAs. Cells were treated with ADNT (**A**) or CONA (**B**) for 72 h and cell viability was assessed by MTT assay. Data from at least three independent experiments performed in triplicates were analyzed. Statistical significance was determined by one-way ANOVA. * *p* < 0.05, ** *p* < 0.005 vs. DMSO-treated cells. # *p* < 0.05 vs. NHAs treated the same way.

Given the high level of PRDX1 and PRDX2 proteins in the majority of GBM tissues, we decided to investigate the effect of the inhibitors of these enzymes on the viability of human GBM cells and NHAs. Treatment with 50 μM of ADNT for 72 h diminished the viability of GBM cells by approximately 20% and NHAs by 60%. When the concentration of ADNT was increased to 100 μM, the viability decreased by 25% in T98G, LUB17, and LUB20 cells, 80% in U87MG and LN229 cells, and 70% in NHAs (Figure 2A).

Treatment of T98G and LUB20 cells with 1 μM of CONA for 72 h diminished their viability by 30%, and U87MG, LN229, and LUB17 cells by 40–50%. The increase in CONA concentration resulted in GBM cell viability being further reduced by 70–90% at 5 μM and 80–90% at 10 μM of CONA. The sensitivity of NHAs to CONA treatment was comparable with that of T98G and LUB20 cells and was lower than the sensitivity displayed by U87MG, LN229, and LUB17 cells (Figure 2B).

Taken together, these results indicate that: (i) a much lower concentration of CONA compared to ADNT is needed to significantly reduce the viability of GBM cells, and (ii) NHAs are equally or less sensitive to CONA treatment than GBM cells. Considering the above, only CONA has been subjected to further studies.

### 3.3. CONA Reduces Clonogenicity of GBM Cells

**Figure 3:**
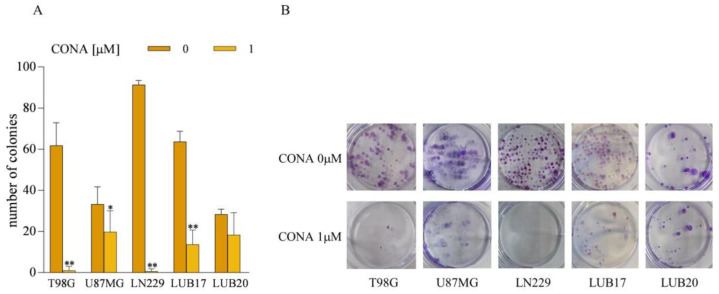
CONA diminishes the clonogenic potential of GBM cells. The cells were treated with CONA for 72 h. Subsequently, the fresh complete medium was added, and the cells were cultured for an additional 10 days. (**A**) Data from three independent experiments. Statistical significance was determined by one-way ANOVA. * *p* < 0.05, ** *p* < 0.005 vs. DMSO-treated cells. (**B**) Representative images of clonogenic assay.

We next investigated the influence of CONA treatment on the ability of GBM cells to form colonies. At concentration of 1 μM of CONA, when added to the medium after the cells were seeded and attached, reduced this parameter in U87MG, LUB17, and LUB20 by 80% and almost completely abolished it in T98G and LN229 cells (Figure 3). No colonies were found in any of the cell lines after treatment with 5 μM of CONA. In the supplementary experimental protocol, the cells were first treated with CONA for 72 h, then counted and plated in the CONA-free medium. In this case, CONA slightly diminished the ability to form colonies, as the number of colonies decreased by 25% when the cells were treated with 10 μM of the compound (Figure S2).

**Figure 4:**
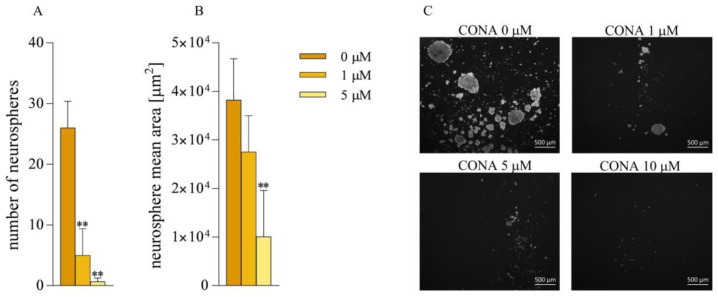
Treatment with CONA reduces neurosphere generation. Quantification analysis of the number (**A**) and mean area (**B**) of neurospheres formed by LUB17N cells cultured with or without CONA for 20 days. ** *p* < 0.005 vs. DMSO-treated cells by one-way ANOVA. (**C**) Representative phase-contrast images (4× magnification) of neurospheres. Bar: 500 μm.

Moreover, treatment with CONA diminished the ability of LUB17N cells to generate neurospheres. Thus, the cells treated with 1 μM of CONA formed a significantly lower number of neurospheres with a slightly smaller mean area compared to their untreated counterparts. Both parameters were further markedly reduced by 5 μM of CONA and completely abolished by 10 μM of CONA (Figure 4).

### 3.4. CONA Induces Autophagosome Accumulation in GBM Cells

**Figure 5:**
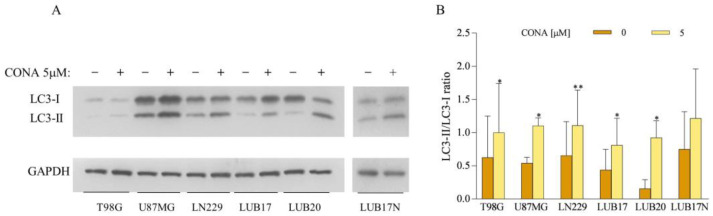
Treatment with CONA increases accumulation of autophagosomes in GBM cells. Western blot analysis of the levels of LC3I and LC3II proteins in the cells treated with DMSO or 5 μM of CONA for 24 h. GAPDH was used as a loading control. (**A**) Representative Western blot images. (**B**) Densitometric analysis of LC3II/LC3I ratio. Experiment was repeated three times. Statistical significance was determined by Student *t* test. * *p* < 0.05, ** *p* < 0.005 vs. DMSO-treated cells.

To investigate the mechanism underlying CONA-induced cell death, we studied the levels of two forms of microtubule-associated protein light chain 3 (LC3). The cytosolic form of LC3, LC3-I, is converted into LC3-II, the protein present on autophagosomes, and therefore serves as an indicator of autophagosome formation. Western blot analysis followed by the quantification of band density revealed that the treatment of GBM cells with 5 μM of CONA for 24 h increased the LC3-II/LC3-I ratio (Figure 5). These results indicate the accumulation of autophagosomes in CONA-treated cells.

### 3.5. ROS Generation Induced by CONA Contributes to GBM Cell Death

**Figure 6:**
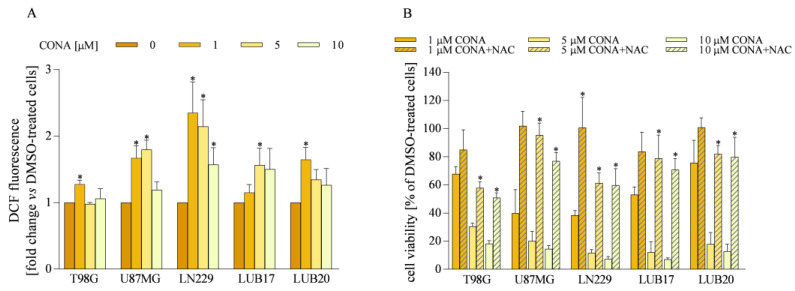
CONA-induced ROS overproduction contributes to the inhibition of cell viability. (**A**) The cells were treated with CONA for 24 h and ROS were detected with a fluorescent probe. (**B**) The cells were treated with CONA for 72 h with or without pre-treatment with 5 mM of NAC for 1 h. Next, cell viability was assessed by MTT assay. Data from three independent experiments performed in triplicates were analyzed. Statistical significance was determined by one-way ANOVA. * *p* < 0.05 vs. DMSO-treated cells (**A**) or not pre-treated with NAC (**B**).

Since CONA has been proved to be an inhibitor of PRDX2 [25], a molecule-reducing ROS, we hypothesized that treatment with CONA enhances ROS formation. Indeed, in all cell lines treatment with CONA increased the ROS level (Figure 6A). The most pronounced elevation in ROS level was found in U87MG and LN229 cells treated with 1 μM and 5 μM of CONA. Pre-treatment with the ROS scavenger NAC prevented CONA-induced cell death (Figure 6B), indicating that ROS overproduction contributes to the reduction of cell viability.

### 3.6. ROS Generators Potentiate the Anticancer Activity of CONA

**Figure 7:**
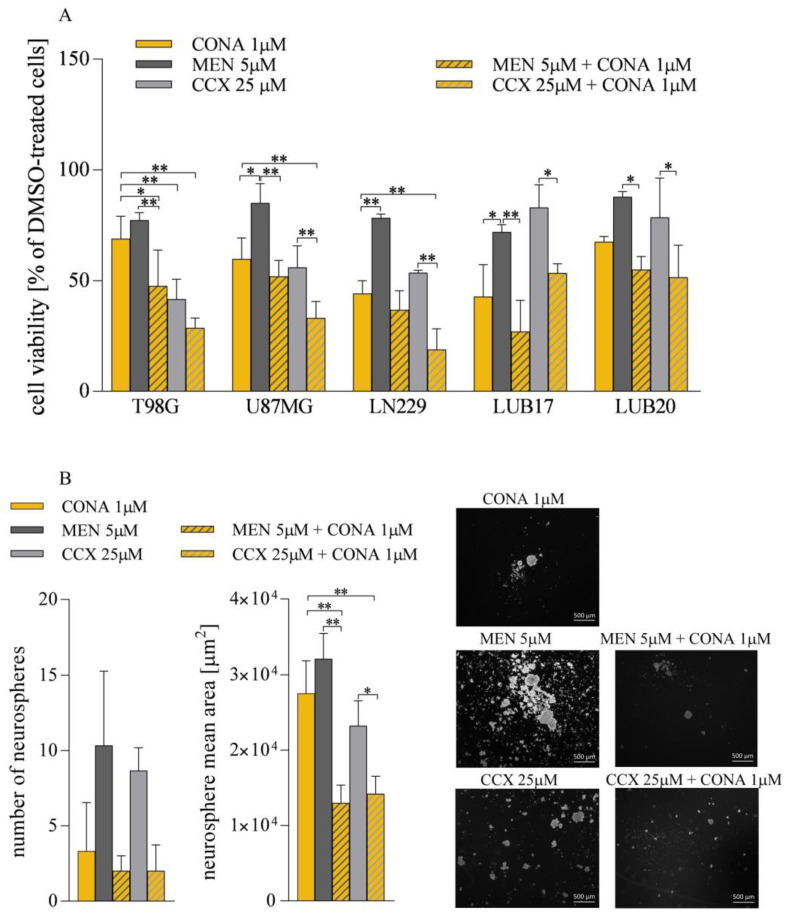
MEN or CCX enhances CONA cytotoxicity in GBM cells. (**A**) The viability of adherent cells treated with either 1 μM of CONA, 5 μM of MEN, and 25 μM of CCX, alone or in different combinations, for 72 h. (**B**) The analysis of the number and mean area of neurospheres generated by LUB17N cells cultured in the presence of 1 μM of CONA, 5 μM of MEN, 25 μM of CCX, or their combinations for 20 days. * *p* < 0.05, ** *p* < 0.005 by one-way ANOVA. Bar: 500 μm.

Based on the previous observation that CONA enhanced ROS formation, we hypothesized that a simultaneous treatment with CONA and ROS inducers would potentiate cell death. To verify this hypothesis, we analyzed the effect of CONA combined with MEN, a synthetic form of vitamin K3, or CCX, a selective cyclooxygenase-2 inhibitor. Both MEN and CCX have previously been shown to induce ROS production [29,30]. Likewise, we observed an elevation in ROS levels in GBM cells treated with 5 μM of MEN or 25 μM of CCX (Figure S3). As shown in Figure 7, when 5 μM MEN was administered alone for 72 h, the cell viability diminished by 15–25%. The combined treatment with 5 μM of MEN and 1 μM of CONA decreased this parameter by 50–70%. Moreover, treatment with 25 μM of CCX reduced the viability by 15–40%, and a further reduction by 50–80% was observed when a combination of 25 μM of CCX and 1 μM of CONA was administered (Figure 7A).

Finally, we analyzed the impact of the combined administration on the ability to generate neurospheres. Both the number of neurospheres and their mean area were much lower in the cells treated with 5 μM of MEN combined with 1 μM of CONA compared to the cells treated with 5 μM of MEN alone. Similarly, simultaneous incubation with 25 μM of CCX and 1 μM of CONA inhibited neurosphere generation more effectively than 25 μM of CCX alone (Figure 7B).

Collectively, the above results indicate that the combination of CONA with either MEN or CCX increases cytotoxicity against GBM cells displayed by each of these compounds when applied individually.

## 4. Discussion

Due to uncontrolled metabolic processes, cancer cells contain high basal levels of ROS. To survive, they stimulate the antioxidant systems, which may lead to resistance to redox-targeted therapies [3,4]. The identification of the mechanisms developed by neoplastic cells to eliminate a surplus of ROS is a crucial step in designing therapies based on oxidative damage.

The primary aim of this study was to examine the level of two antioxidant enzymes, PRDX1 and PRDX2, in GBM. A tendency toward increased *PRDX1* and *PRDX2* expression found in GBM compared to NT tissues is in line with the results of analysis of publicly available datasets [31]. Moreover, our finding of an elevated level of PRDX1 protein in GBM is consistent with a previous report documenting the upregulation of PRDX1 in a few GBM cases compared to peritumoral tissues [23]. Regarding PRDX2, there are no data comparing its level between GBM and NT tissues so far, although one study showed higher PRDX2 levels in GBM cells than in astrocytes and an immortalized glial cell line [26]. Hence, this is the first report documenting an increased PRDX2 protein level in GBM compared to non-neoplastic brain tissue.

High amounts of PRDX1 and/or PRDX2 and their growth-supporting functions have been documented in several cancer types [12,32,33], suggesting that inhibitors of these enzymes may exert anticancer activity. Here, we examined the influence of ADNT, a compound which has been shown to inhibit the antioxidant activity of PRDX1 and, to a lesser extent, PRDX2 [18], on the viability of GBM cells. At a 50 μM concentration, ADNT decreased the viability of neoplastic cells by 20%, while the viability of NHAs was diminished by 60%. An increase in ADNT concentration to 100 μM caused a further decrease in the viability of both GBM cells and NHAs. Given the lower level of PRDX1 in NHAs compared to GBM cells, one would expect the lower sensitivity of astrocytes to PRDX1 inhibition. However, the sensitivity of NHAs to 100 μM of ADNT was comparable to that of the U87MG and LN229 cells, but significantly higher than that of T98G and the patient-derived GBM cells LUB17 and LUB20. This particularly high sensitivity of NHAs to ADNT may suggest a critical function of PRDX1 in astrocyte survival. Indeed, its expression was induced in reactive astrocytes around the hemorrhagic region [34] and was shown to be associated with astrocyte proliferation after spinal cord injury [35].

The other compound whose anticancer activity we examined was CONA, a covalent inhibitor of PRDX2 [20]. The viability of GBM cells diminished by 30–50% when they were treated with 1 μM of CONA, and further decreased by 70–90% when 5 μM of CONA was used. At both concentrations, NHAs were as sensitive as T98G and LUB20 GBM cells, and less sensitive than U87MG, LN229, and LUB17 cells. The reasons for this phenomenon remain unknown, but one may speculate that PRDX2 plays a particularly important role in the survival of these three GBM cell lines. It is worth mentioning that the LN229 cells turned out to be the most vulnarable to the CONA administration in a long-term clonogenic assay, in which treatment with 1 μM of CONA completely abolished their ability to form colonies. Surprisingly, T98G cells also displayed very high susceptibility to the CONA treatment in this assay, although they were relatively resistant to 1 μM of CONA in the MTT test. Among the adherent cells used in this study, patient-derived LUB20 cells appeared to be the most resistant to CONA, which could result from the considerable amount of PRDX2 detected in these cells. A profound inter- and intratumoral heterogeneity is a hallmark of GBM [36,37], which is thought to contribute to the differential response to treatment [38,39], and most likely accounts for the discrepancy in the response to CONA treatment observed in our study. Indeed, variation in the sensitivity to ROS-producing therapies has been linked to the heterogeneous level of antioxidant enzymes [40] or the status of the genes encoding isocitrate dehydrogenases (IDHs) 1 and 2 [41]. Furthermore, the *IDH1* mutation or amplification of the gene encoding the epidermal growth factor receptor (EGFR) resulted in the overproduction of ROS, triggering severe oxidative stress [42,43] and modulated cell susceptibility to targeted therapies [41].

It should be mentioned that in order to analyze the influence of CONA on the ability to form colonies, we used two essentially different protocols. In the first protocol, which is often used for screening for the sensitivity of cells to different treatments, cells are plated before treatment. In the second one, which is used especially in radiobiological research to determine lethal and sublethal damage repair, cells are first treated and subsequently re-plated [44]. The results we obtained indicate that treatment with CONA significantly reduces the ability of GBM cells to form colonies, but cells that manage to survive such treatment do not lose their clonogenic potential.

Interestingly, patient-derived cells grown in neurospheres, LUB17N, formed single neurospheres after treatment with 5 μM of CONA, suggesting that they are less susceptible to this compound compared with their counterparts grown as an adherent monolayer. This feature may be related to the very high level of PRDX2, and is consistent with a general assumption that GBM spheroids adapt to the elevated intracellular ROS levels by enhancing their protective antioxidant system. Indeed, it was demonstrated that GBM adherent cells were more susceptible to radiation and presented higher radiation-induced oxidative stress compared to GBM spheroids [45]. Moreover, Van Loenhout et al. showed that adherent GBM cells were more sensitive to auranofin (AF), an inhibitor of antioxidant thioredoxin reductase, than the relevant cells cultured in neurospheres [46]. Similarly, in our previous study, LUB17N cells showed increased resistance to AF compared to their counterparts cultured as an adherent monolayer [47].

CONA treatment enhanced the generation of ROS in all GBM cells examined. It is noteworthy that ROS overproduction was most pronounced in LN229 and U87MG cells, which turned out to be the most susceptible to the short-term CONA administration. Furthermore, pre-treatment with a ROS scavenger, NAC, protected cells from CONA-induced death, indicating the crucial role that an excess of ROS plays in the reduction in cell survival. This finding prompted us to analyze the effects of CONA applied in combination with MEN or CCX, whose ability to induce ROS production in cancer cells has been previously described [29,30]. Here, we show that monotherapy with either 5 μM of MEN or 25 μM of CCX reduces the viability of GBM cells only slightly or moderately. However, this parameter is dramatically reduced when the combined treatment with either CONA and MEN or CONA and CCX is used. Additionally, both combinations lead to a high toxicity in GBM neurospheres.

It should be highlighted that, so far, only a few papers have documented the anticancer activity of CONA [21,48]; therefore, the current state of our knowledge on the molecular mechanisms underlying anticancer properties of this compound is limited. Our results clearly indicate that CONA-induced ROS production contributes to the reduction in GBM cell viability. However, one cannot exclude that its mode of action goes beyond the perturbation of redox homeostasis. Additionally, although CONA has been identified as a covalent PRDX2 inhibitor, it could potentially affect the function of the other PRDX isoforms. To prove that this compound decreases GBM cell viability through the inhibition of PRDX2, it would be necessary to compare the influence of CONA on the phenotype of the wild type and either the *PRDX2*-silenced cells or the cells containing mutated PRDX2 that are unable to bind CONA. Furthermore, an increased LC3II isoform level in CONA-treated cells allows us to speculate that this compound triggers autophagy, but further detailed studies are required to determine to what extent this process contributes to CONA-induced cell death. Moreover, since a few studies have tested CONA in different in vivo models, data on its safety are limited. Recently, the intraventricular injection of CONA was shown to attenuate lysed erythrocyte-induced hydrocephalus, ventricle wall damage, and inflammatory responses, and no adverse effects of CONA were observed [49]. Furthermore, a CONA co-injection diminished brain swelling, neuronal death, and neurological deficits caused by the intracerebral injection of lysed erythrocytes [50]. Nevertheless, the question about the ability of CONA to cross the blood–brain barrier, one of the major obstacles for the treatment of brain tumors, remains unanswered.

## 5. Conclusions

This study shows the upregulation of PRDX1 and PRDX2 antioxidant enzymes in GBM compared to non-tumor brain tissues. Furthermore, our work demonstrates the cytotoxic effect of CONA, a PRDX2 inhibitor, in GBM cells grown both as a monolayer or as neurospheres. Moreover, this compound enhances ROS production, which contributes to CONA-mediated cell death. Cytotoxic properties of CONA are markedly potentiated by MEN or CCX, which are ROS-inducing agents. Altogether, our results encourage carrying out further studies aimed at testing the efficiency of CONA and other novel PRDX1/2 inhibitors alone or in combination with ROS-inducing drugs in GBM models.

## Data Availability

The data presented in this study are available from the corresponding author upon reasonable request.

## Supplementary Materials

The following supporting information can be downloaded at: https://www.mdpi.com/article/10.3390/cells12151934/s1, Figure S1: Western blot analysis of the level of PRDX1 and PRDX2 proteins in NT (n = 20) and GBM (n = 41) tissues; Figure S2: The number of colonies formed by GBM cells treated with CONA according to the supplementary protocol; Figure S3: Intracellular DCF fluorescence intensity of the cells treated with either 5 mM MEN or 25 mM CCX for 24 h.
