# Supplementary figures and images for: Conoidin A, a Covalent Inhibitor of Peroxiredoxin 2, Reduces Growth of Glioblastoma Cells by Triggering ROS Production
Source: Cells. 2023 Jul 26;12(15):1934. doi: 10.3390/cells12151934 (PMC10417327; doi:10.3390/cells12151934)

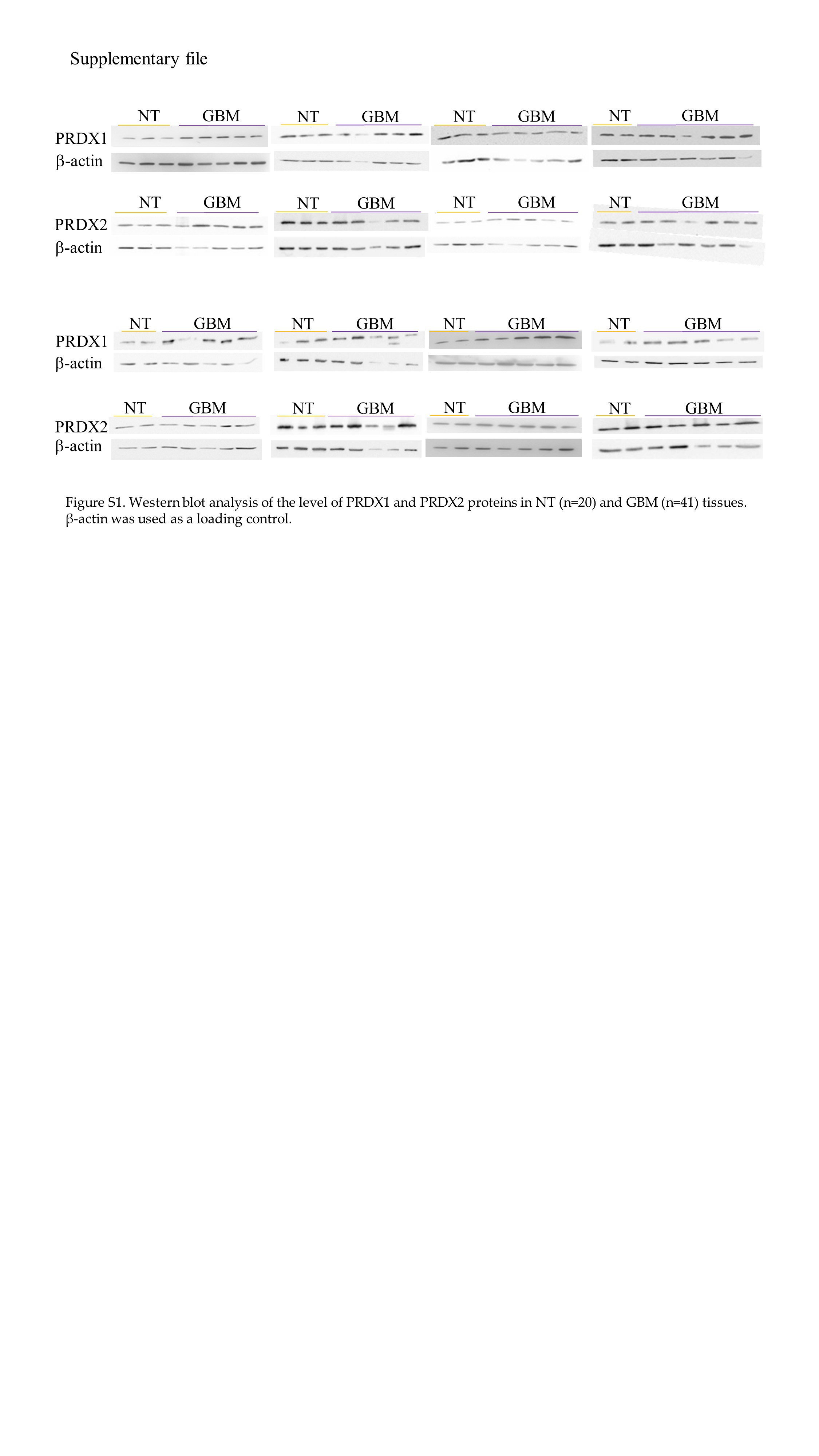

Supplement: Supplementary file 1 [file cells-12-01934-s001.zip › Figure S1.jpg]

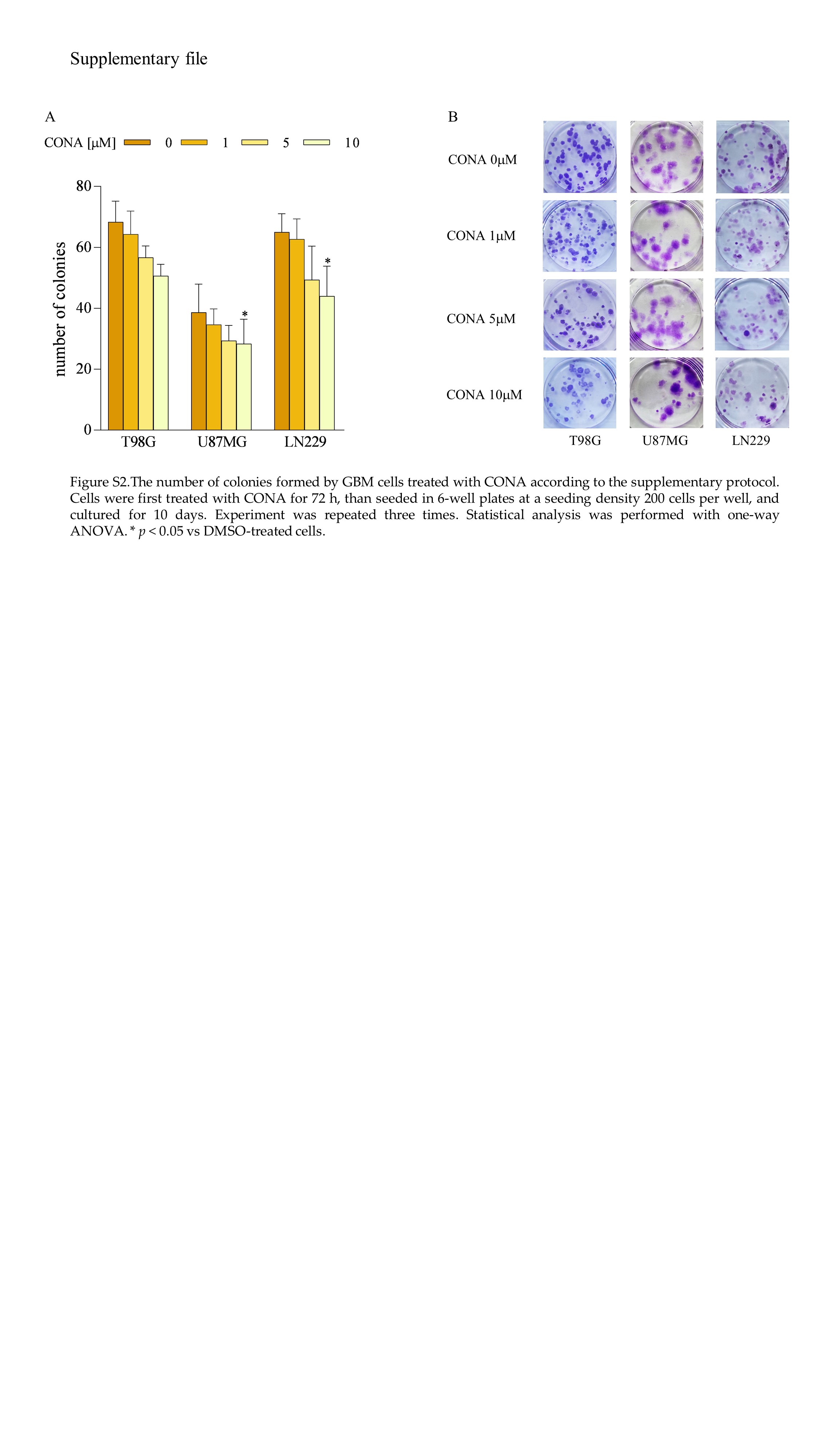

Supplement: Supplementary file 1 [file cells-12-01934-s001.zip › Figure S2.jpg]

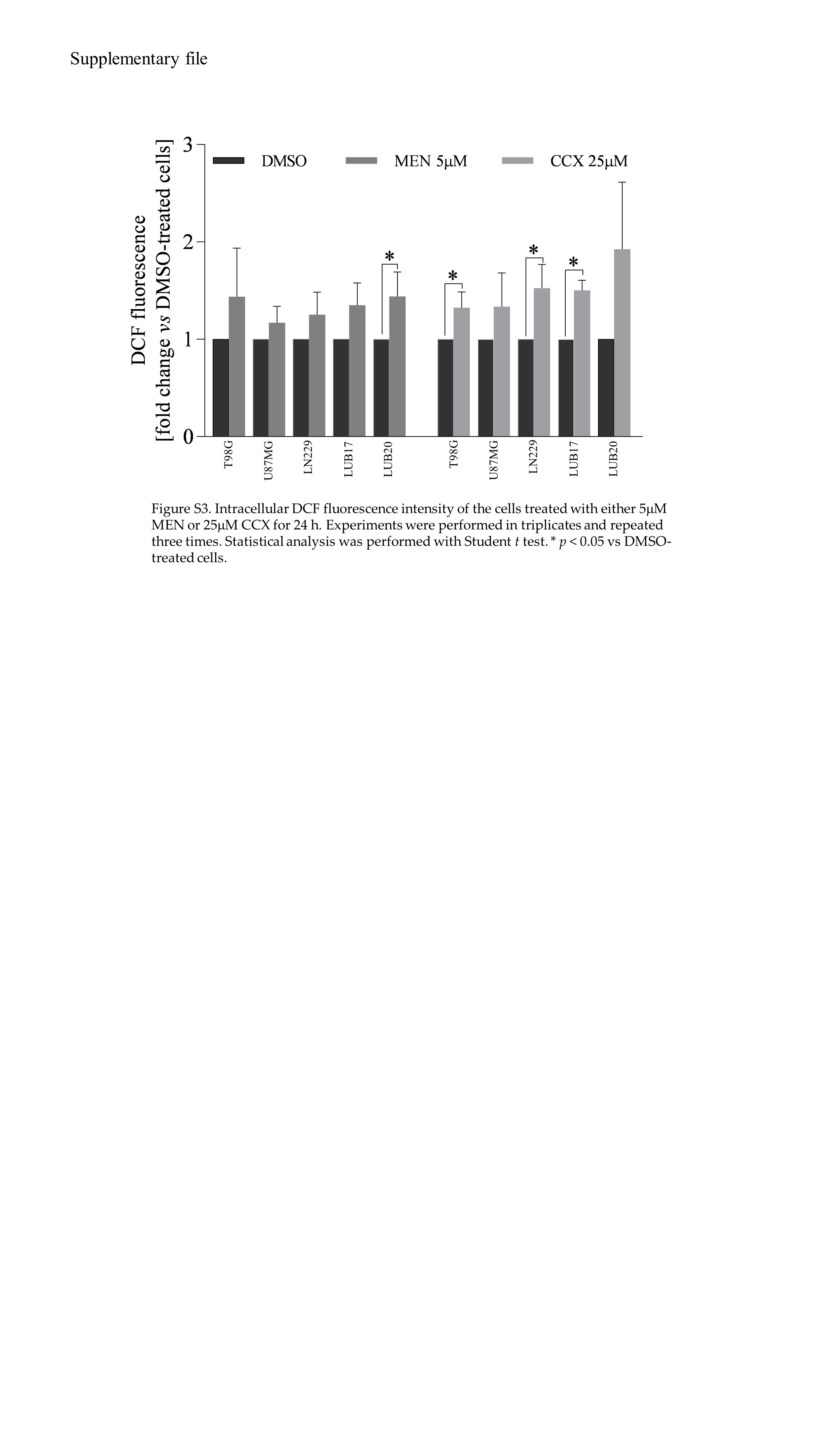

Supplement: Supplementary file 1 [file cells-12-01934-s001.zip › Figure S3.jpg]
